# Early Carcinogenesis Involves the Establishment of Immune Privilege via Intrinsic and Extrinsic Regulation of Indoleamine 2,3-dioxygenase-1: Translational Implications in Cancer Immunotherapy

**DOI:** 10.3389/fimmu.2014.00438

**Published:** 2014-10-06

**Authors:** Alisha Holtzhausen, Fei Zhao, Kathy S. Evans, Brent A. Hanks

**Affiliations:** ^1^Department of Pharmacology and Cell Biology, Duke University Medical Center, Durham, NC, USA; ^2^Division of Medical Oncology, Department of Medicine, Duke University Medical Center, Durham, NC, USA

**Keywords:** indoleamine 2,3-dioxygenase, dendritic cells, tumor immune evasion, β-catenin, tumor immunotherapy, Wnt5a, type III TGF-β receptor, COX-2

## Abstract

Although prolonged genetic pressure has been conjectured to be necessary for the eventual development of tumor immune evasion mechanisms, recent work is demonstrating that early genetic mutations are capable of moonlighting as both intrinsic and extrinsic modulators of the tumor immune microenvironment. The indoleamine 2,3-dioxygenase-1 (IDO) immunoregulatory enzyme is emerging as a key player in tumor-mediated immune tolerance. While loss of the tumor suppressor, BIN-1, and the over-expression of cyclooxygenase-2 have been implicated in intrinsic regulation of IDO, recent findings have demonstrated the loss of TβRIII and the upregulation of Wnt5a by developing cancers to play a role in the extrinsic control of IDO activity by local dendritic cell populations residing within tumor and tumor-draining lymph node tissues. Together, these genetic changes are capable of modulating paracrine signaling pathways in the early stages of carcinogenesis to establish a site of immune privilege by promoting the differentiation and activation of local regulatory T cells. Additional investigation of these immune evasion pathways promises to provide opportunities for the development of novel strategies to synergistically enhance the efficacy of the evolving class of T cell-targeted “checkpoint” inhibitors.

## Introduction

**Figure 1:**
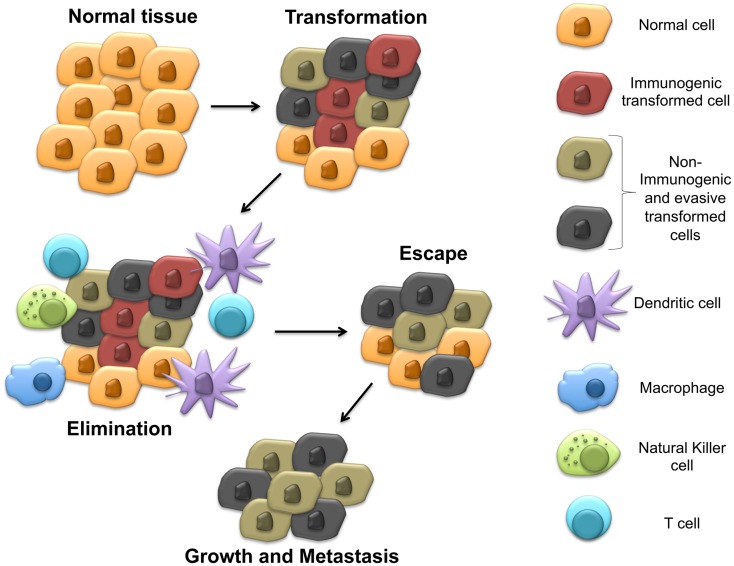
**Early steps in carcinogenesis include immune evasion by upregulating IDO expression in the tumor microenvironment**. This model proposes the development of immune evasion mechanisms early during transformation, which stimulate local IDO activity prior to the development of adaptive anti-tumor immunity and generation of the selective pressure responsible for cancer immunoediting.

Indoleamine 2,3-dioxygenase-1 (IDO) is a heme-containing enzyme known to catalyze the rate limiting step in the degradation of the essential amino acid tryptophan to its metabolic byproducts known collectively as the kynurenines (1). Although initially felt to play primarily an anti-microbial role, pioneering work eventually showed this biochemical pathway to impact the immune system by inhibiting T cell proliferation and driving the differentiation and activation of regulatory T cells (Tregs) (2–6). While IDO has been broadly implicated in the progression of several cancers by aiding tumors to evade the host immune system, the mechanisms utilized by cancers to regulate IDO activity have remained poorly characterized (7, 8). Recent work in pre-clinical models has revealed novel mechanisms utilized by cancers to manipulate IDO activity within the tumor microenvironment. Interestingly, these mechanisms have been found to be regulated by previously defined tumor suppressors and oncogenes, which undergo genetic alteration relatively early during tumorigenesis. In contrast to the cancer immunoediting hypothesis, which proposes that immune-mediated selective pressure by the adaptive immune system is necessary before active immune tolerization mechanisms develop (9), this observation suggests that subversion of the immune system is necessary at relatively early stages of tumor development and, in fact, occur concurrently with the process of malignant transformation (Figure 1). This review discusses our understanding of IDO regulation, highlights mechanisms utilized by cancers to control IDO activity in the tumor immune microenvironment, and outlines pharmacological strategies for reversing these processes to ultimately augment our immunotherapeutic strategies for managing cancer patients.

## Regulation of IDO Expression and Enzymatic Activity

Several factors have been shown to regulate the expression of IDO in a cell type-specific manner in monocytes, macrophages, endothelial cells, fibroblasts, some tumor cells, and various populations of dendritic cells (DCs). Initial studies focused on the process of inflammation, showing interferon-γ (IFN-γ) to be a potent inducer of IDO expression in many cell types and demonstrating this pathway of IDO induction to provide protection from intracellular pathogens by depleting local tryptophan levels (10, 11). Other inflammatory factors that have been shown to regulate IDO expression include IL-1, tumor necrosis factor-α (TNF-α), and lipopolysaccharide (LPS) (12, 13). Interestingly, prostaglandin E2 has been observed to induce the transcription of IDO in human monocyte-derived DCs; however, additional activation with TNF-α or LPS was noted to be necessary to achieve full enzymatic activity (14). This is consistent with observations by other investigators who have found that two-signals are often necessary to induce maximal IDO expression by specific DC subsets (5). Additional T cell-derived signals have also been demonstrated to play a role in regulating IDO expression including reverse signaling via B7 co-stimulatory molecule (CD80/CD86) cross-linking on the surface of antigen-presenting cells (APCs) (15–18). Using this mechanism, Tregs constitutively expressing CTLA-4 condition DCs by stimulating IDO expression and, in turn, suppressing local T cell proliferation, thereby establishing a state of immune tolerance. Later work revealed this B7 reverse signaling mechanism to be dependent upon the activation of the non-canonical NFκB signaling pathway, a mechanism also responsible for the induction of IDO following stimulation by other cell surface receptors including CD40 and the glucocorticoid-induced TNF receptor (GITR) (19).

It has been generally proposed that IDO activation signals have evolved to provide a negative feedback mechanism to dampen local inflammatory processes and prevent immune-mediated pathology. However, more recent studies have shown the immunosuppressive cytokine, TGF-β, to induce novel IDO functionality by specific subtypes of DCs including the murine CD8^+^ splenic DC subset as well as the plasmacytoid DC (pDC) population (20, 21). These studies revealed TGF-β to stimulate the expression and Fyn-dependent phosphorylation of IDO, enabling this protein to also serve as a scaffolding molecule for downstream signaling ultimately leading to the expression of both TGF-β and IDO itself. As opposed to the rapid and short-lived induction of IDO expression by IFN-γ, the stimulation of IDO expression by TGF-β is thought to be durable and to serve as a mechanism for generating long-term immune tolerance. This post-translational modification of IDO by TGF-β also has additional implications in terms of its regulation. In inflammatory conditions, exposure to IL-6 promotes the degradation of IDO by upregulating suppressor of cytokine signaling 3 (SOCS3), which binds to a phosphorylated immunoreceptor tyrosine-based inhibitory motif (ITIM) in IDO and promotes its proteosomal degradation (22).

Additional post-translational regulatory mechanisms have been elucidated, which are also capable of contributing to the regulation of IDO activity. This includes the nitration of various IDO tyrosine residues by peroxynitrite, a byproduct of nitric oxide (NO) and superoxide, which has also been shown to dampen IDO enzyme activity in macrophages (23). This is consistent with other findings showing NO to directly inhibit IDO activity by binding to its active site heme moiety (24). Indeed, previous work indicates that the reduction–oxidation status of the cell is capable of modulating activity of the IDO holoenzyme by interfering with the heme biosynthetic pathway (25).

It is clear that there are multiple mechanisms, which may regulate IDO on both the transcriptional and post-translational levels. However, the biological contexts in which these regulatory mechanisms affect IDO activity remain unclear. This is particularly true for the process of carcinogenesis, which occurs in a biochemically altered environment. Several studies have supported an important role for IDO in the generation of an immunotolerant tumor microenvironment that facilitates tumor progression (26–28). These findings indicate that the mechanisms utilized by cancers to modulate IDO expression and activity may be central to understanding the highly complex process of carcinogenesis. Here, we discuss recent studies investigating the mechanisms that cancers utilize to manipulate local IDO activity within the immune microenvironment in order to facilitate their metastatic progression.

## Tumor-Mediated Regulation of Intrinsic IDO1 Expression

**Figure 2:**
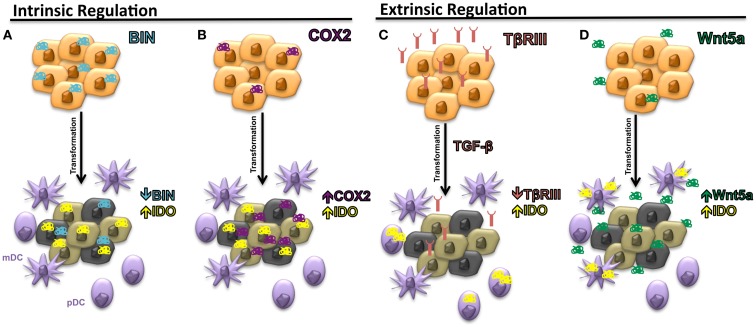
**Intrinsic and extrinsic mechanisms of IDO regulation in the tumor microenvironment**. **(A)** Downregulation of *Bin1* expression leads to enhanced expression of IDO by tumor cells. **(B)** Upregulation of cyclooxygenase-2 (COX-2) expression by tumor cells stimulates intrinsic tumor expression of IDO. **(C)** Loss of *T*β*RIII*, the gene encoding the type IIII TGF-β receptor (TβRIII), allows for increased TGF-β paracrine signaling in the tumor microenvironment and the upregulation of IDO by local plasmacytoid DCs (pDCs). **(D)** Increased soluble Wnt5a secretion upregulates IDO expression by local myeloid DCs (mDCs).

The expression of IDO by many cancer types has been correlated with inferior progression-free and overall survival (29). However, the regulation of IDO expression by malignant tissues has been poorly understood. In 2005, the first mechanism by which several solid tumors can regulate the intrinsic expression of IDO was described. This work focused on the BAR adapter-encoding gene BIN-1, a tumor suppressor previously found to be downregulated in several transformed cell lines and demonstrated to suppress the transformational activity of MYC by interacting with its N-terminus (30). Subsequent studies revealed BIN-1 to interfere with malignant transformation utilizing several mechanisms beyond the suppression of MYC activity and additional work showed BIN-1 to play the role of a tumor suppressor in several cancer types including melanoma, breast cancer, colon cancer, and prostate cancer (31–33). After noting that BIN-1 seemed to suppress the development of a transformed epithelial tumor model via an immune-dependent mechanism, Muller and colleagues noted that the deletion of *Bin-1* significantly enhanced the IFN-γ-mediated upregulation of IDO expression by tumor cells (34). Indeed, the observed enhancement in tumor growth following *Bin-1* deletion was reversed in the presence of the 1-methyltryptophan (1-MT) IDO inhibitor only in the setting of an intact immune system. These authors concluded that BIN-1 was capable of modulating IDO expression by regulating the STAT1 and NFκB signaling pathways that have been previously implicated in promoting the transcription of *Indo*, the IDO encoding gene (Figure 2A). This represented the initial study linking IDO regulation to an intrinsic tumor suppressor pathway by showing that the loss of *Bin1* tumor expression contributes to tumorigenesis by driving cellular proliferation while simultaneously concealing itself from detection and destruction by the host immune system. This work prompted us to conjecture that early phases of tumor initiation and growth will often require the evolution of multifunctional genes, which regulate both cell division and/or survival, as well as elements of the local immune microenvironment.

Cyclooxygenase-2 (COX2), another modulator of IDO expression and activity, has also been implicated in the process of tumorigenesis and may be consistent with this hypothesis (35). COX2 represents the inducible isoform of the cyclooxygenases and plays a critical role in eicosanoid biosynthesis, including the prostaglandins and leukotrienes. The initial data linking COX2 biology with carcinogenesis was provided by genetic studies showing that when APC^Δ716^ mice, which model the colon cancer syndrome, familial adenomatous polyposis, are crossed with mice carrying an inactivating mutation in the *Pgst2* gene encoding COX2, offspring develop exhibiting a diminished number of intestinal polyps (36). Since this study, several pre-malignant and malignant tissues have been shown to express COX2 at relatively early time points of tumorigenesis and several pro-tumorigenic functions have been ascribed to COX2 including the promotion of tumor-mediated angiogenesis, anti-apoptosis, and the generation of the epidermal growth factor receptor ligand, amphiregulin (37). One of the downstream products of COX2 activity, prostaglandin E2 (PGE2), has been previously demonstrated to interfere with T cell and DC function (38). Additional studies have shown COX2 and IDO expression to correlate in both human breast cancer cell lines and human breast cancer primary tissues while other investigators have found PGE2 to directly stimulate IDO expression (14, 39). Interestingly, COX2 inhibitors enhance the anti-tumor effects of DC-based vaccines and promote tumor-specific T cell responses in the MMTV-*neu* autochthonous murine mammary carcinoma model further suggesting an immunologic role for COX2 in cancer (35). Further studies have also shown COX2 inhibitors to augment a MUC1-based vaccine in a transgenic pancreatic cancer model in a manner that depended on suppressed IDO activity within tumor tissues (40). Similar roles for COX2 in promoting Tregs in non-small cell lung cancer and in elevating IDO expression in acute myeloid leukemia have also been described (41, 42). Together, these studies suggest that COX2 represents an important regulator of IDO function within malignant tissues (Figure 2B). While these studies focused on investigating the relationship between COX2 expression and the intrinsic regulation of IDO expression by tumor cells, a more recent study has shown a COX2-expressing MCF-7 breast cancer cell line to induce IDO expression by co-cultured fibroblasts, suggesting that paracrine IDO regulatory networks may also be relevant during the process of carcinogenesis (43).

## Characterizing Tumor-Mediated Regulation of Extrinsic IDO1 Expression

Although it is unclear if the cell type expressing the IDO enzyme may affect its ultimate immunologic impact in the setting of cancer, previous investigators have shown a relationship between local DC expression of IDO and poor clinical prognosis in patients with melanoma (28, 44). In light of these data, we reasoned that tumor-derived soluble factors may have evolved to manipulate local DC expression of this critical immune regulatory mechanism. Further, in light of the dual role of the BIN-1 tumor suppressor described above, we searched for soluble factors already described to have a pro-tumorigenic role in the literature. These criteria led us to the type III TGF-β receptor (TβRIII) that functions as a co-receptor for the TGF-β signaling pathway by binding and presenting all three TGF-β isoforms to the type I and II TGF-β receptor signaling complex (45). TβRIII has been independently implicated in suppressing cellular migration in several experimental systems through a β-arrestin2-mediated mechanism (46, 47). Consistent with the criteria discussed above, earlier studies also revealed TβRIII to be shed at the cell surface and for its soluble form, sTβRIII, to bind and suppress downstream TGF-β-mediated signaling, effectively functioning as a molecular sink for the TGF-β cytokine (48, 49). Additional work has demonstrated human breast cancers to downregulate TβRIII expression by loss of heterozygosity and for TβRIII to impede metastatic progression of the 4T1 murine breast cancer model (50). This loss of TβRIII expression has since been shown to occur during the progression of several additional cancers including pancreatic, lung, and prostate cancers (51–53). Notably, the downregulation of TβRIII has also been demonstrated to occur at a relatively early stage of tumorigenesis, exemplified by the loss of TβRIII in tissue specimens histologically characterized as ductal carcinoma *in situ*, an early precursor to invasive ductal carcinoma of the breast. Additional studies showing the loss of TβRIII to promote the epithelial-to-mesenchymal transition (EMT) in pancreatic cancer further suggests that this genetic alteration occurs at a relatively early time point during carcinogenesis (53).

TGF-β has been described as a pleiotropic regulator of the immune system capable of modulating both IDO activity and several additional immunosuppressive pathways (54). By inhibiting both T cell and NK cell proliferation and activation, as well as promoting the differentiation of Treg populations, the TGF-β cytokine plays an important role in maintaining peripheral immune tolerance. We, therefore, hypothesized that the loss of TβRIII expression by developing malignancies would allow for enhanced TGF-β-dependent signaling in the tumor microenvironment, thereby inhibiting local immune surveillance mechanisms and ultimately promoting tumorigenesis (55). Using the 4T1 murine breast cancer model, we initially determined that TβRIII expression suppressed metastatic progression only in immunocompetent hosts. Consistent with this observation, further work revealed the loss of TβRIII to be associated with the development of an immunotolerant microenvironment characterized by a decrease in the number of CD8^+^ T cells and a corresponding increase in the CD4^+^FoxP3^+^ Treg population in both breast cancer and melanoma model systems.

These findings led to the identification of an association between TβRIII expression and suppressed levels of IDO in both the tumor bed and within the tumor-draining lymph nodes (TDLNs). We determined that the loss of TβRIII expression correlated with the upregulation of IDO by pDC populations within TDLN tissues, the same cell population that was previously noted to be important for the expression of IDO within TDLN tissues (Figure 2C) (21, 27). These results also corresponded with diminished pDC and whole TDLN tissue IDO enzyme activity when recovered from mice bearing TβRIII-expressing tumors. Since sTβRIII is an effective inhibitor of downstream TGF-β signaling, we hypothesized that TGF-β was a major mediator of pDC IDO activity in the tumor microenvironment and confirmed the findings of Pallotta and colleagues by showing TGF-β treatment of purified pDCs to enhance IDO expression and enzymatic activity (21). We then demonstrated this paracrine signaling mechanism to be functionally relevant by demonstrating purified pDCs derived from mice harboring TβRIII-downregulated tumors, to suppress *in vitro* T cell proliferation in an IDO-dependent manner. Further, using a doxycycline-inducible system, we showed that the earlier the alteration in TβRIII expression by a developing tumor, the more profound the effect on the local tumor microenvironment. These results suggest that modulation of the immune microenvironment at earlier stages of tumorigenesis is associated with a greater impact on tumor progression.

The work described above illustrates the potential for genetic alterations within the tumor to impact local DC function and to subvert immunosurveillance. Given the critical role that these APCs play in orchestrating the anti-tumor immune response, it follows that the evolution of various mechanisms to convert local DCs into a tolerogenic state would be quite advantageous for a developing tumor. The pathways for driving DC tolerogenesis in the setting of cancer remain poorly characterized; however, the β-catenin signaling pathway has emerged as a potentially important component of this process. This is supported by data showing that activation of the β-catenin pathway in myeloid DCs (mDCs) conditioned these APCs to promote the generation of IL-10-expressing CD4^+^ T cells capable of suppressing the autoimmune phenotype of a mouse model of multiple sclerosis (56). These findings were further substantiated by *in vivo* experiments using the CD11c-cre × β-cat^lox/lox^ transgenic model demonstrating the DC-specific β-catenin pathway as an important regulator of Treg differentiation in gastrointestinal tissues (57). These studies raised the possibility that tumors may promote DC tolerization in the tumor microenvironment via the expression of soluble Wnt ligands. Interestingly, several members of the Wnt ligand family have been noted to play a role in carcinogenesis. Indeed, mechanistic studies have revealed Wnt5a to promote melanoma cell migration and invasion, ultimately leading to disease metastases (58). In addition, increased Wnt5a expression levels in melanoma tissues as well as diminished levels of the soluble Wnt antagonist, Dkk-1, have been associated with an inferior clinical outcome in patients with advanced melanoma (59–63).

Together, the above reports led us to screen the conditioned media of several human melanoma cell lines for their ability to stimulate downstream β-catenin signaling activity. This work consistently showed that melanoma-derived conditioned media was capable of inducing this signaling pathway in both reporter cell lines and primary DCs *in vitro* (64). Further work using the *Tyr:CreER;Braf^CA^;Pten^lox/lox^* inducible transgenic melanoma model, showed that TDLN DCs and tumor-infiltrating DCs associated with developing melanomas expressed elevated levels of known β-catenin target genes including *Axin2*, *Ccnd1*, *C-myc*, and *Tcf-7* relative to DCs derived from more distant lymph node tissues. This local paracrine β-catenin signaling effect was then confirmed *in situ* within the melanoma stroma and within TDLN tissues by confocal microscopy using the Tg(TCF/Lef1-HIST1H2BB/EGFP)61Hadj/J transgenic reporter strain, which encodes an EGFP reporter downstream of a β-catenin-responsive promoter containing tandem TCF/LEF1 transcription factor binding elements (65).

After verifying that this signaling pathway could be induced within mDCs in an autochthonous model of melanoma, we demonstrated that β-catenin was promoting tolerogenic DC development by regulating the downstream expression of IDO. Although recent studies had shown Wnt-mediated signaling to stimulate expression of the immunosuppressive factor IL-10 by DCs, the effects on IDO expression were unknown (61). Using a variety of methods including pharmacological inhibitors, promoter–reporter systems, immunofluorescence microscopy, and chromatin immunoprecipitation, we were able to demonstrate that Wnt ligands robustly induced the durable upregulation of both IDO expression and enzymatic activity by bone marrow-derived DCs in a β-catenin-dependent manner. By performing blocking experiments, we further noted that the soluble Wnt5a ligand was the dominant factor in melanoma-conditioned media in the induction of IDO by DCs and that Wnt5a was capable of promoting IDO expression in an IFN-γ-independent manner (Figure 2D). Importantly, as opposed to several other stimuli including Wnt3a, additional work revealed Wnt5a conditioned DCs to significantly promote the differentiation and expansion of naïve CD4^+^ T cells into Tregs in an IDO-dependent manner. Indeed, further *in silico* gene expression analysis has revealed a significant association between *Wnt5a* and *Foxp3* gene expression levels in human melanomas. Similar to BIN-1, COX2, and TβRIII above, this work implicates Wnt5a as a factor with dual roles in carcinogenesis including tumor invasion and metastasis, as well as the suppression of local immune surveillance. Similar to TβRIII, Wnt5a has also been implicated in the promotion of EMT in both pancreatic cancer and gastric cancer (66, 67). This finding implies that Wnt5a is upregulated and is capable of modulating the immune microenvironment at an early time point during the process of carcinogenesis suggesting that Wnt5a likely has an impact on the establishment of immune privilege during the earliest stages of transformation.

## Therapeutic Implications of IDO Regulatory Pathways in Cancer-Mediated Immune Evasion

Although recent advances in immunotherapy have made substantial strides in improving clinical responses in patients with advanced cancers, a significant fraction of these patients continue to fail therapy. In light of the diverse array of immune evasion mechanisms that individual cancers are able to employ to escape detection and destruction by the host immune system, it seems that combinatorial therapies, which target different aspects of immune suppression will be necessary to fully realize the promise of immunotherapy. While the majority of immunotherapy development has targeted T cell-expressed negative regulators such as CTLA-4 and PD-1 (68, 69), few agents are currently under investigation, which are capable of modulating tolerogenic DCs in the tumor microenvironment. An exception to this includes the IDO inhibitor, 1-methyltryptophan, which was shown to enhance chemotherapeutic effects in both the murine orthotopic 4T1 and the autochthonous MMTV-*neu* breast cancer models (70). Given these encouraging findings, high throughput screening has been employed to identify improved compounds for further clinical trial development (71–73). Although single agent efficacy has been modest (74), recent reports are showing encouraging clinical responses when combined with anti-CTLA-4 monoclonal antibody therapy while several other combination studies are ongoing (75–79) (Table 1). These results suggest that immunotherapeutic regimens employing a combinatorial approach including T cell-targeted immune checkpoint inhibitors and agents capable of reversing tumor-mediated immune evasion mechanisms have great promise.

**Table 1:** **Active clinical trials investigating the activity of IDO inhibitors in advanced cancer**.

Agent	Regimen	Disease	Sponsor	ClinicalTrials.gov Identifier	Phase of development	Reference
NLG-919	Monotherapy	Advanced solid tumors	New link genetics	NCT02048709	I	(72)
Indoximod	Temozolomide	Glioblastoma multiforme	New link genetics	NCT02052648	I/II	(75)
Indoximod	Docetaxel	Breast cancer	New link genetics	NCT01792050	II	(76)
Indoximod	Sipuleucel-T	Prostate cancer	New link genetics	NCT01560923	II	(77)
Indoximod	Ipilimumab	Melanoma	New link genetics	NCT02073123	I/II	(78)
INCB024360	Ipilimumab	Melanoma	Incyte Corp.	NCT01604889	I/II	(74)
INCB024360	Pembrolizumab	Lung cancer and other solid tumors	Incyte Corp./Merck & Co.	NCT02178722	I/II	clinicalTrials.gov
INCB024360	Anti-DEC-205/NY-ESO-1 vaccine and poly-ICLC	Ovarian, fallopian, peritoneal cancers	Fred Hutchinson Cancer Center/CITN/Celldex Therapeutics	NCT02166905	I/II	clinicalTrials.gov
INCB024360	MELITAC multipeptide vaccine	Melanoma	Fred Hutchinson Cancer Research Center/CITN/Incyte Corp.	NCT01961115	II	clinicalTrials.gov

*Ipilimumab, anti-CTLA-4 monoclonal antibody (Bristol-Myers Squibb). Pembrolizumab, anti-PD-1 monoclonal antibody (Merck & Co.). anti-DEC-205-NY-ESO-1, DC-targeted antibody-peptide fusion vaccine (CDX-1401, Celldex Therapeutics). Poly-ICLC, polyinosinic-polycytidylic acid and poly-l-lysine double-stranded RNA TLR3 agonist (Oncovir, Inc.). MELITAC multipeptide vaccine, emulsion of a mixture of 12 class I MHC-restricted melanoma peptides (University of Virginia), CITN, Cancer Immunotherapy Network*.

**Figure 3:**
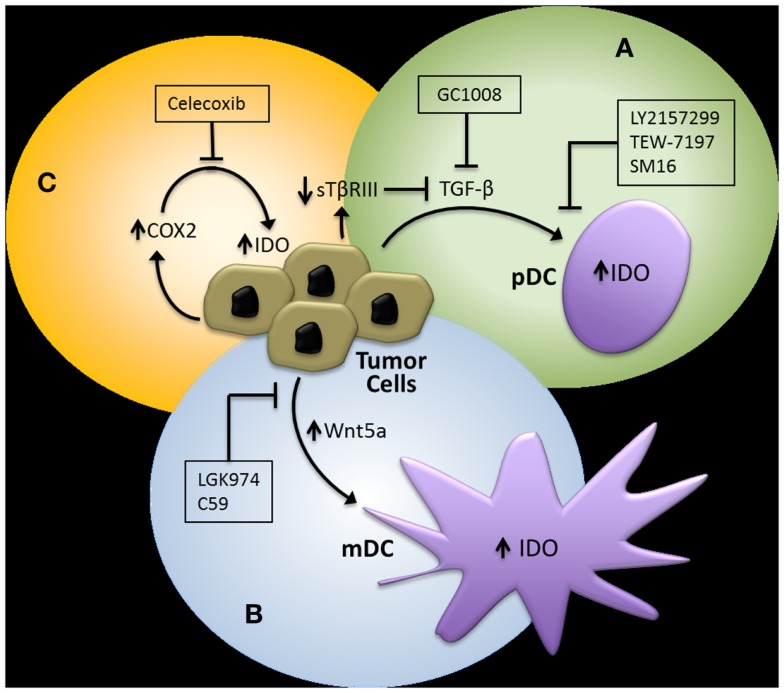
**Potential pharmacological strategies for suppressing IDO expression in the tumor microenvironment**. **(A)** TGF-β inhibitors. GC1008, pan-TGF-β isoform monoclonal antibody (Genzyme), LY2157299, type I TGF-β receptor serine/threonine kinase small molecule inhibitor (Eli Lilly), TEW-7197, type I TGF-β receptor serine/threonine kinase small molecule inhibitor (MedPacto, Inc.). **(B)** Wnt inhibitors. LGK974, Porcn acyl transferase small molecule inhibitor (Novartis). **(C)** Cyclooxygenase-2 (COX2) Inhibitors. Celecoxib (Pfizer, Inc.). pDC, plasmacytoid DC; mDC, myeloid DC.

In addition to targeting the IDO immunoregulatory enzyme itself, pharmacological strategies designed to interfere with the previously discussed regulatory pathways of IDO have theoretical advantages (Figure 3). First, evidence showing that TGF-β-induced IDO in specific DC subsets is capable of maintaining immune tolerance via a signaling mechanism that is independent of its enzymatic activity suggests that inhibitors targeting the active site of IDO may have limited clinical benefit (21). Second, IDO is likely to only be a component of the tolerogenic DC program induced by specific tumor-derived mediators, implying that inhibition of the upstream signals that activate this program are more likely to have greater clinical efficacy. This is exemplified by TGF-β, which is known to induce other immunosuppressive pathways involving a variety of cellular targets (54).

Since our studies have indicated that tumor-derived TGF-β is capable of inducing IDO expression by pDCs and that this effect is enhanced upon loss of TβRIII in a murine breast cancer model, we investigated the ability of an oral type I TGF-β receptor serine/threonine kinase inhibitor (SM16) to augment the immunologic response of a Her2/neu vaccine. This work confirmed that a type I TGF-β receptor serine/threonine kinase inhibitor could synergistically enhance the CD8^+^ T cell host immune response to Her2/neu and effectively suppress the progression of a Her2/neu-expressing 4T1 breast cancer model (55). Given these findings as well as the supporting data suggesting that the loss of TβRIII in murine melanoma models also promotes the development of an immune suppressive microenvironment, we investigated the combination of another oral type I TGF-β receptor kinase inhibitor currently in clinical trial development, LY2157299 monohydrate (80, 81), with anti-CTLA-4 monoclonal antibody therapy in the *Tyr:CreER;Braf^CA^;Pten^lox/lox^* transgenic melanoma mouse model (82). Consistent with our previous findings, this combinatorial treatment approach also generated a synergistic anti-tumor response, effectively enhancing the tumor-infiltrating CD8^+^ T cell/Treg ratio and suppressing both primary melanoma development and the establishment of distant metastasis.

As described previously, emerging data suggest that the tumor-mediated expression of Wnt5a contributes to the generation of an immunotolerant microenvironment. We, therefore, hypothesized that the inhibition of Wnt5a-mediated signaling would also augment immunotherapy efficacy in melanoma. Several strategies to inhibit the Wnt-β-catenin pathway have been investigated; however, one of the more promising approaches is targeting the membrane-bound *O*-acyltransferase, Porcn, which catalyzes the palmitoylation of all vertebrate Wnt ligands, a step necessary for effective secretion of the Wnt soluble protein family (83, 84). This work has led to the introduction of LGK974, a small molecule Porcn acyltransferase inhibitor, into early phase clinical trials (85). To determine if the inhibition of Wnt secretion by targeting Porcn would be an effective approach for reversing melanoma-mediated immune suppression, we genetically silenced Porcn expression by the B16 murine melanoma model and performed several *in vivo* tumor assays. This new B16-PORCN^KD^ cell line was found to exhibit impaired Wnt secretion, suppressed tumor growth *in vivo*, and for this to be associated with both enhanced levels of infiltrating CD8^+^ T cells and suppressed levels of PD-1-expressing tumor-infiltrating lymphocytes. With this data, we then evaluated the ability of a commercially available pharmacological inhibitor of Porcn, C59, to reverse B16-mediated immune suppression (86). Given as monotherapy to mice bearing B16 melanomas, this agent did not seem to exhibit a significant anti-tumor effect, however, when combined with anti-CTLA-4 monoclonal antibody therapy, a synergistic enhancement in activated 41BB^+^ TRP2-specific CD8^+^ tumor-infiltrating lymphocytes were observed along with B16 tumor growth suppression. Together, these data support the use of combinatorial immunotherapy strategies that involve agents capable of interfering with tumor immune evasion pathways including the upregulation of local IDO expression.

## Conclusion

Studies focused on understanding the interplay between tumor development and the host immune system are now revealing an intimate relationship between the processes of tumor invasion and metastasis and the active induction of immune tolerance. Rather than developing as a response to immune-mediated selective pressure, we hypothesize that some immune evasion mechanisms are capable of developing at a very early stage in carcinogenesis and simultaneously promoting tumor invasion while also interfering with tumor detection by the host immune system. The pathways that we have found to meet these criteria are induced by intrinsic genetic alterations, resulting in the downregulation of both the BIN-1 and TβRIII tumor suppressors and the upregulation of the pro-tumorigenic factors, COX2 and Wnt5a. Interestingly, this body of work also highlights important differences in cell-specific IDO expression kinetics. While IFN-γ is a rapid inducer of IDO expression in many cell types, studies are now demonstrating that the loss of *T*β*RIII* in a TGF-β^hi^ tumor microenvironment promotes durable IDO expression by pDCs while the upregulation of the Wnt5a oncogene results in durable IDO expression by mDC populations (Figures 2C,D). We expect for several other tumor-mediated soluble factors or perhaps exosome-derived factors to also regulate IDO expression via similar paracrine signaling mechanisms. Their identification will be important for therapeutic development as well as for the establishment of predictive biomarkers to determine when these novel therapeutic strategies would be most effectively employed. Importantly, pre-clinical experimental investigation, to date, suggests that the use of a pharmacologic agent to inhibit these tumor-mediated evasion pathways which target IDO activity effectively synergize with immune checkpoint blockade. These data strongly support the physiologic relevance of these novel immune evasion pathways, which target IDO activity within the tumor microenvironment.

## Conflict of Interest Statement

The authors declare that the research was conducted in the absence of any commercial or financial relationships that could be construed as a potential conflict of interest.
